# Concomitant Immune Checkpoint Inhibitors Induced Central and Primary Adrenal Insufficiency: A Diagnostic Challenge

**DOI:** 10.1210/jcemcr/luaf194

**Published:** 2025-08-22

**Authors:** Alberto Nascè, Sophie Leboulleux, Simina Chiriac, Petros Tsantoulis, Maria Mavromati

**Affiliations:** Department of Endocrinology and Diabetology, Hôpitaux Universitaires de Genève, Geneva 1205, Switzerland; Department of Endocrinology and Diabetology, Hôpitaux Universitaires de Genève, Geneva 1205, Switzerland; Department of Radiology and Medical Informatics, Hôpitaux Universitaires de Genève, Geneva 1205, Switzerland; Department of Medical Oncology, Hôpitaux Universitaires de Genève, Geneva 1205, Switzerland; Department of Endocrinology and Diabetology, Hôpitaux Universitaires de Genève, Geneva 1205, Switzerland

**Keywords:** immune checkpoint inhibitors, hypophysitis, adrenalitis

## Abstract

Immune checkpoint inhibitors (ICI) have several endocrine toxicities. Dual sequential pituitary and adrenal toxicities are rare and difficult to diagnose. This case report describes a 63-year-old man with metastatic clear cell renal carcinoma treated with combined nivolumab and ipilimumab. After 3 cycles, he presented with severe headaches. The imaging studies revealed pituitary enlargement consistent with hypophysitis. Hormonal assessment confirmed panhypopituitarism, leading to high-dose hydrocortisone replacement. Four months later, a routine computed tomography scan showed significant bilateral adrenal enlargement. Later symptoms of fatigue and low blood pressure, combined with high potassium, suppressed aldosterone, and high renin, confirmed the diagnosis of immune checkpoint inhibitor-associated adrenalitis, while ACTH remained low due to pituitary function impairment. Fludrocortisone was added, resulting in clinical improvement, and a control scan showed reduced adrenal size compared to previous imaging. This case emphasizes the importance of radiological and laboratory assessments in diagnosing concomitant central and peripheral adrenal insufficiency during ICI therapy.

## Introduction

Immune checkpoint inhibitors (ICIs) have improved cancer survival by harnessing immune responses against tumors but may cause endocrine complications. This clinical case highlights the rare dual toxicity of pituitary and adrenal glands from combined nivolumab and ipilimumab treatment, emphasizing diagnostic challenges and the importance of imaging and laboratory findings.

## Case Presentation

The patient was a 63-year-old man known for delusional disorder, chronic obstructive pulmonary disease, and stage III obesity. His treatment included inhaled β-agonists and corticosteroids and liraglutide. He had a history of left nephrectomy for clear renal cell carcinoma staged pT1b cN0 cM0. Three years later, pulmonary metastases and mediastinal metastatic lymph nodes were diagnosed. He started combined ipilimumab [an anti-cytotoxic T-lymphocyte antigen 4 (anti-CTLA-4)] and nivolumab [an anti-programmed cell death protein-1 (anti-PD-1)] every 3 weeks.

**Figure 1.**
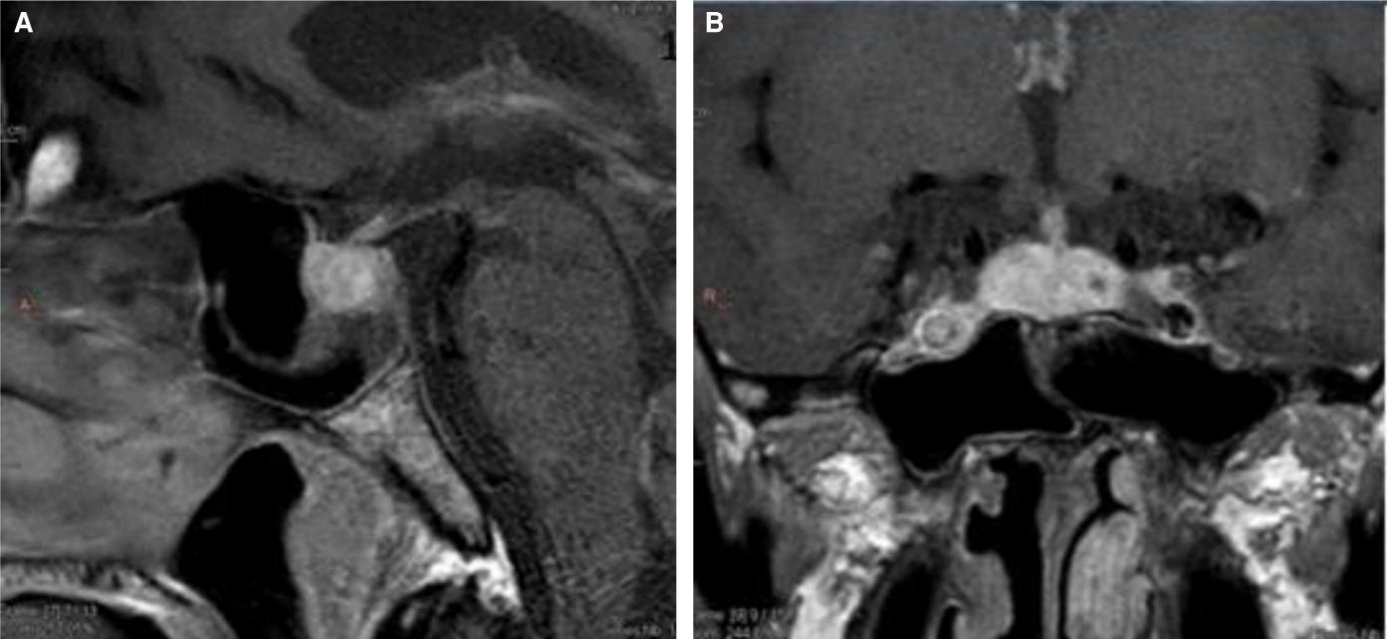
T1-weighted pituitary magnetic resonance imaging. (A) (sagittal view) and (B) (frontal view), taken at hypophysitis diagnosis, show global enlargement of the pituitary gland and heterogeneous enhancement after gadolinium injection.

After 3 cycles, he presented with severe retro-orbital headaches. Brain magnetic resonance imaging (MRI) showed global pituitary enlargement, with a convex superior aspect, and intense, slightly heterogeneous enhancement after gadolinium injection (Fig. 1A and 1B). The findings indicated immune checkpoint inhibitor-associated hypophysitis (ICI-aH), prompting high-dose hydrocortisone therapy. Hormonal investigations revealed panhypopituitarism with central hypothyroidism, hypogonadism, and GH deficiency (Table 1). Thyroid ultrasonography and antithyroid peroxidase antibodies were not assessed because of the central origin of hypothyroidism. Levothyroxine was started, while gonadal therapy was deferred due to the lack of symptoms. Immunotherapy was discontinued due to severe endocrine toxicity.

**Table 1.** Hormonal check-up on immune checkpoint inhibitors-associated hypophysitis diagnosis

Parameter tested	Value	Reference range
TSH	0.021 mIU/L (SI: 0.021 mIU/L)	0.27-4.2 mIU/L (SI: 0.27-4.2 mIU/L)
Free T4	0.49 ng/dL (SI: 6.3 pmol/L)	0.93-1.71 ng/dL (SI: 12-22 pmol/L)
Free T3	2.3 pg/mL (SI: 3.6 pmol/L)	2-4.4 pg/mL (SI: 3.1-6.8 pmol/L)
FSH	6.6 mIU/mL (SI: 6.6 IU/L)	1.5-12.4 mIU/mL (1.5-12.4 IU/L)
LH	4.6 mIU/mL (SI: 4.6 IU/L)	1.7-8.6 mIU/mL (1.7-8.6 IU/L)
Testosterone	74 ng/dL (SI: 2.56 nmol/L)	273-816 ng/dL (SI: 9.46-28.29 nmol/L)
SHBG	2.92 µg/mL (SI: 26 nmol/L)	2.36-7.98 µg/mL (SI: 21-71 nmol/L)
IGF-1	50 ng/mL (SI: 6.5 nmol/L)	49-188 ng/mL (SI: 6.37-24.44 nmol/L))
ACTH	NA	7-63 ng/L (SI: 1.54-13.8 pmol/L)
Cortisol	NA	—
Prolactin	7.3 ng/mL (SI: 7.3 µg/L)	4-15.2 ng/mL (SI: 4-15.2 µg/L)

Abbreviations: NA, not available; SI, international system of units.

## Diagnostic Assessment

**Figure 2.**
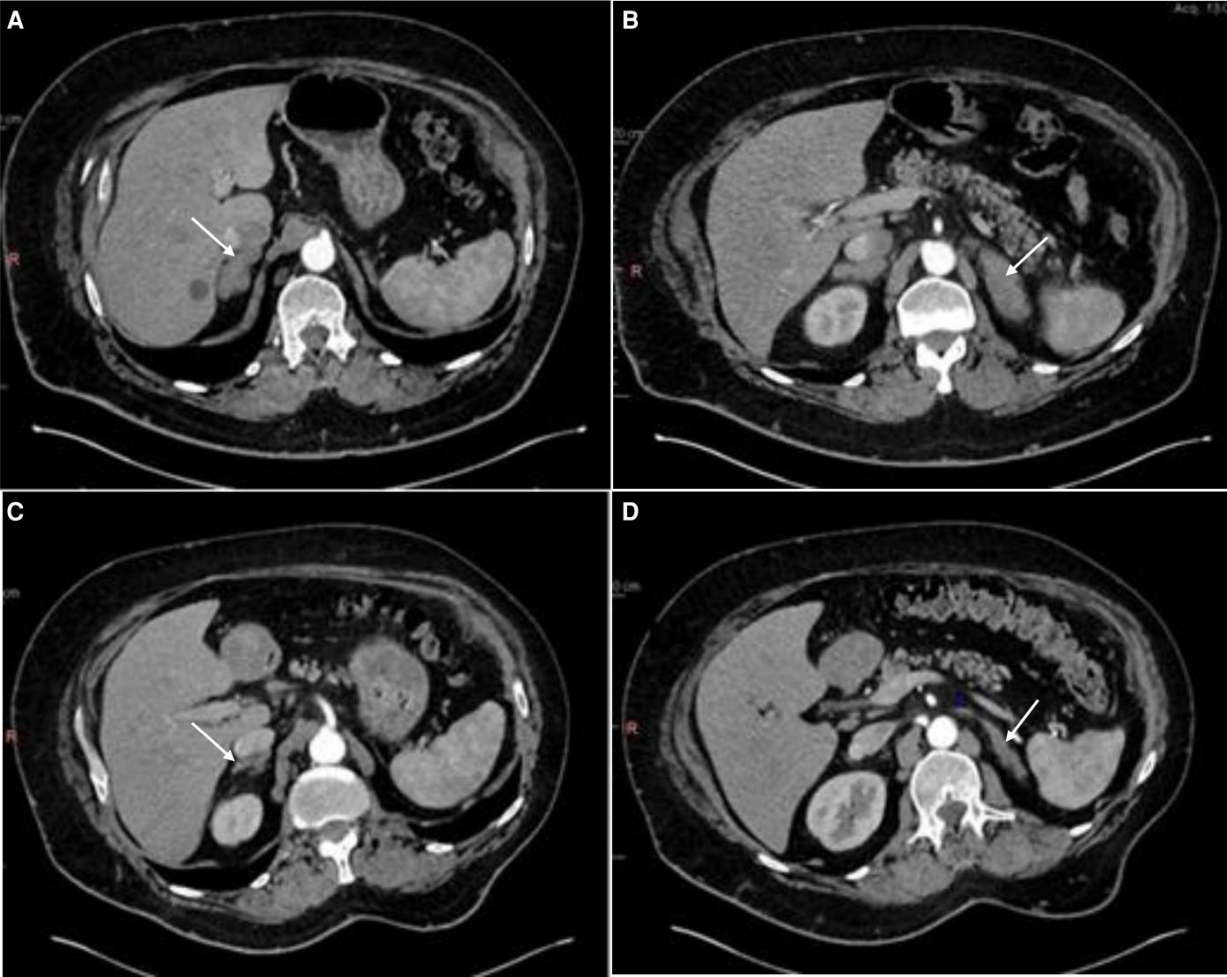
Contrast CT scan shows adrenal thickening of the right (A) and left (B) adrenal consistent with immune checkpoint inhibitors-associated adrenalitis. (C) and (D) show adrenal thickening resolution. White arrows highlight both adrenal glands. CT scans were performed on a 128-slice machine with an arterial time acquisition [start of acquisition 10 seconds from the optimal enhancement of the region of interest (at 100 Hounsfield Units) at the upper abdominal level and a venous phase thoraco-abdominopelvic acquisition (venous phase: 30 seconds from the end of arterial acquisition)]. Abbreviation: CT, computed tomography.

Four months later, the headaches resolved. Central adrenal insufficiency was confirmed with low basal cortisol, low ACTH, and low plasma cortisol after cosyntropin stimulation test (Table 2). Radiological assessment showed stable disease but new bilateral adrenal enlargement [right adrenal gland: 23 mm thick, with a density of 32 Hounsfield Units (HU) on the arterial and 46HU on the venous phase; left adrenal gland: 10 mm thick, with a density of 31HU on the arterial and 55HU on the venous phase] (Fig. 2A and 2B). Tumor infiltration and adrenalitis were possible diagnoses. Spontaneous density was unavailable due to contrasted computed tomography (CT) scan. The patient was asymptomatic and missed several medical appointments.

**Table 2.** Cosyntropin stimulation test confirming central hypocortisolism

Parameter tested	Value	Reference range
ACTH	<2 ng/L (<0.44 pmol/L)	7-63 ng/L (SI: 1.54-13.8 pmol/L)
Cortisol T0	3.62 ng/mL (SI: 10 nmol/L)	—
Cortisol T1	24.65 ng/mL (SI: 68 nmol/L)	—

Abbreviations: NA, not available; SI, international system of units.

Two months later he reported fatigue, initially attributed to a 60 kg weight loss (30% of body weight) over 1 year on liraglutide. He had hypotension (90/60 mmHg) and tachycardia (100 bpm), despite increased hydrocortisone from 20 to 40 mg/day. Laboratory workup revealed hyperkalemia, suppressed aldosterone, elevated renin, and low androgens (Table 3). A CT scan showed reduced adrenal size (Fig. 2C and 2D), ruling out neoplasia: right gland 6 mm (28 HU arterial, 48 HU venous) and left gland 6 mm (28 HU arterial, 36 HU venous). Diagnosis of immune checkpoint inhibitor-associated adrenalitis (ICI-aA) was retained and fludrocortisone started.

**Table 3.** Hormonal workup on immune checkpoint inhibitors-associated adrenalitis diagnosis

Parameter tested	Value	Reference range
ACTH	<2 ng/L (< 0.44 pmol/L)	7-63 ng/L (SI: 1.54-13.8 pmol/L)
Cortisol	131.95 ng/mL (SI: 364 nmol/L)	—
Potassium	5.2 mEq/L (SI: 5.2 mmol/L)	3.6-4.6 mEq/L (SI: 3.6-4.6 mmol/L)
Renin (lying)	6.9 ng/mL/h (SI: 6.9 ng/mL/h)	0.2-2.8 ng/mL/h (SI: 0.2-2.8 ng/mL/h)
Aldosterone (lying)	<2.52 ng/dL (SI: <70.1 pmol/L)	5.32-41.62 ng/dL (SI: 147.8-1154.6 pmol/L)
DHEA-S	129 µg/L (SI: 3.48 µmol/L)	517-2950 µg/L (SI: 13.9-79.6 µmol/L)
Androstenedione	8.82 ng/dL (SI: 0.3 nmol/L)	61.7-279.4 ng/dL (SI: 2.1-9.5 nmol/L)

Abbreviations: DHEA-S, dehydroepiandrosterone sulfate; SI, international system of units.

## Treatment

Upon ICI-aH diagnosis, high-dose hydrocortisone was initiated (20 mg 3 times a day) and nivolumab 3 mg/kg and ipilimumab 1 mg/kg were suspended due to clinical severity and the patient's request.

Levothyroxine (0.075 mg/day) was prescribed; gonadal replacement was deferred.

Six months later, worsening fatigue, hypotension, and tachycardia prompted his general practitioner to double hydrocortisone doses (progressively tapered from 60 mg/day within a few weeks). Due to persistent symptoms and new findings supporting an ICI-aA diagnosis, fludrocortisone (0.05 mg/day) was added and hydrocortisone reduced to physiological doses (20 mg/day).

## Outcome and Follow-up

**Figure 3.**
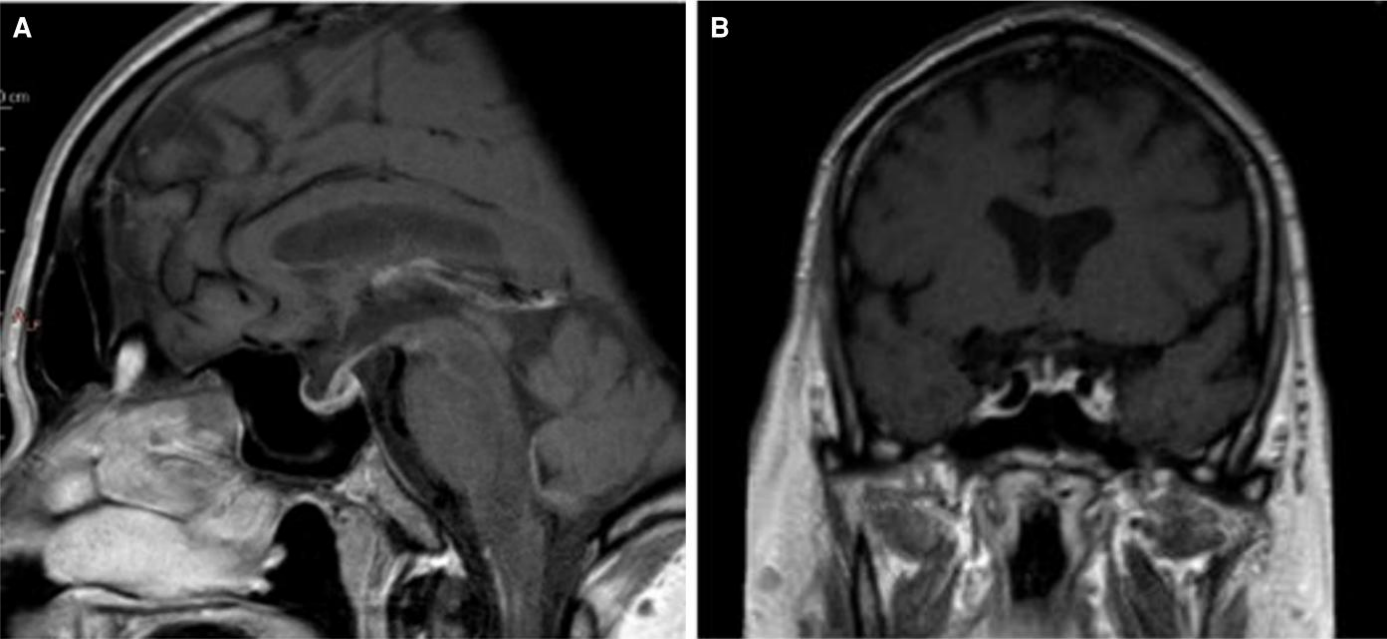
T1-weighted pituitary magnetic resonance imaging. (A) (sagittal view) and (B) (frontal view) show a clear reduction in pituitary gland inflammation.

After treatment modification, symptoms improved. 21-α-hydroxylase autoantibodies were negative. MRI showed reduced pituitary volume (from 10 to 3 mm) (Fig. 3A and 3B).

At the last endocrinology visit, the patient reported good general condition. Vitals were normal. Gonadal axis normalized, and thyroid function remained stable on levothyroxine (Table 4). He continued both glucocorticoid and mineralocorticoid replacement. His renal carcinoma showed a deep partial response 14 months after ICI discontinuation.

**Table 4.** Last hormonal workup, performed under levothyroxine treatment, 14 months after ICI-aA diagnosis, showing gonadotropic axis recuperation

Parameter tested	Value	Reference range
TSH	0.91 mIU/L (SI: 0.91 mIU/L)	0.27-4.2 mIU/L (SI: 0.27-4.2 mIU/L)
Free T4	1.22 ng/dL (SI: 15.8 pmol/L)	0.93-1.71 ng/dL (SI: 12-22 pmol/L)
Free T3	2.58 pg/mL (SI: 3.97 pmol/L)	2-4.4 pg/mL (SI: 3.1-6.8 pmol/L)
FSH	11.7 mIU/mL (SI: 11.7 IU/L)	1.5-12.4 mIU/mL (1.5-12.4 IU/L)
LH	7.3 mIU/mL (SI: 7.3 IU/L)	1.7-8.6 mIU/mL (1.7-8.6 IU/L)
Testosterone	285 ng/dL (SI: 9.88 nmol/L)	273-816 ng/dL (SI: 9.46-28.29 nmol/L)
SHBG	4.87 µg/mL (SI: 43.3 nmol/L)	2.36-7.98 µg/mL (SI: 21-71 nmol/L)
IGF-1	33 ng/mL (SI: 4.31 nmol/L)	49-188 ng/mL (SI: 6.37-24.44 nmol/L)
Prolactin	11.6 ng/mL (SI: 11.6 µg/L)	4-15.2 ng/mL (SI: 4-15.2 µg/L)
Potassium	4 mEq/L (SI: 4 mmol/L)	3.6-4.6 mEq/L (SI: 3.6-4.6 mmol/L)

Cortisol, adrenocorticotropic hormone, renin, and aldosterone are not interpretable since the patient self-administered hydrocortisone and fludrocortisone before the test.

Abbreviations: ICI-aA, immune checkpoint inhibitor-associated adrenalitis; SI, international system of units.

## Discussion

Endocrinopathies are frequent ICI-related adverse events, primarily affecting pituitary and thyroid glands [1]. Symptoms, from mild to life-threatening, may arise at ICI initiation, during treatment, or even after discontinuation [2-4].

We present a rare case of rapid-onset severe hypophysitis followed by adrenalitis induced by ipilimumab and nivolumab combination administered for metastatic clear cell renal carcinoma. In large clinical trials, like Checkmate 214, high-grade hypophysitis and all-cause adrenal insufficiency were rare [5], yet cohort studies highlight a higher prevalence of ICI-related pituitary dysfunctions (13.7-19% with anti-CTLA-4/anti-PD-1 combination therapy; 1-6% in monotherapies) [6, 7]. Diagnosis can be challenging; our focus is on imaging's critical role to support clinical and biological suspicion of these 2 ICI-related endocrinopathies.

Like our patient, most affected individuals are male and over 60 [6, 7]. Median onset is 2.8 months after treatment initiating anti-CTLA-4/anti-PD-1 combination therapy and 4.1 months with monotherapies [6]. Hormonal deficits vary by ICI type. Central adrenal deficit is encountered in most ICI-aH patients [6, 7]. CTLA-4-induced hypophysitis shows ACTH deficiency in 95%, central hypothyroidism in 85%, and hypogonadism in 75% of cases [8]. Anti-PD-1-induced hypophysitis shows isolated ACTH deficiency in more than 90% [8].

Symptoms are often nonspecific, and diagnosis can be difficult in tumoral contexts [8]. Our patient had severe headaches, common in ICI-aH during ipilimumab/nivolumab treatment [6], but no visual or hypopituitarism symptoms. We confirmed ICI-aH by laboratory findings and imaging. Differential diagnoses for pituitary enlargement include metastases and adenomas; MRI helps differentiate between them [6-10]. MRI abnormalities include an initial gland enlargement, returning to normal within weeks or a few months [11]. Gadolinium-enhanced MRI typically shows enhancement of the posterior portion of the pituitary gland, which is homogeneous in 63% of cases and heterogeneous in 37%, including our patient [11]. The pituitary stalk is thickened in 59% of ICI-aH cases and normal in 41%, including in our patient [11]. However, normal imaging does not exclude pituitary impairment [6, 12]. MRI abnormalities are more common with anti-CTLA-4 or anti-CTLA-4/anti-PD-1 therapies [6, 7, 11].

As recommended by European guidelines, our patient received high-dose hydrocortisone due to neurocompressive symptoms. There is no proven benefit of supraphysiological corticosteroid doses in reversing hypopituitarism [13]. Immunotherapy was discontinued due to symptom severity and patient preference.

Primary adrenal insufficiency is a very rare ICI-related endocrine complication, seen in 1.9% of patients on anti-CTLA-4/anti-PD-1 therapy and in 1% on monotherapies [4]. With some PD-1 and PD-L1 inhibitors, an autoimmune-mediated mechanism has been suggested as the underlying cause, with some patients showing 21-hydroxylase autoantibodies [14, 15]. In the absence of concomitant hypophysitis, diagnosis is based on paired low morning cortisol levels and ACTH levels greater than twice the upper limit of normal range [16]. Diagnosing primary adrenal insufficiency in patients already treated with glucocorticoid replacement for central adrenal insufficiency can be challenging; ACTH levels are not necessarily expected to rise in as pituitary function is already abolished [16]. Clinical features slightly differ between primary and secondary adrenal insufficiency. Weight loss (70-100%), nausea/vomiting (60%), and hypotension (70%) are more common in patients with primary adrenal insufficiency where glucocorticoid deficiency is combined with mineralocorticoid deficiency [17]. Salt craving (40-60%), hyperpigmentation (40-70%), and hyponatremia (70-80%) are typical of primary adrenal insufficiency [17]. Aldosterone deficiency leads to renal potassium retention and hyperkalemia, not seen in central adrenal insufficiency [17]. We could, therefore, argue that the difficulty of reducing hydrocortisone to physiologic doses due to recurrent hypotension and hyperkalemia should raise the suspicion for a combined cortisol and aldosterone deficiency in patients at risk, such as those treated with ICI, even if ACTH is normal or low. Our patient showed severe fatigue despite hydrocortisone supraphysiological doses. ACTH remained suppressed due to ICI-aH and the possible contribution of chronic inhaled corticoids and recently added oral hydrocortisone. In this context, ACTH is unreliable to diagnose ICI-aA. An interesting case report presented a patient with triple endocrine dysfunction (thyroiditis, central and peripheral adrenal insufficiency) and showed a significant increase in ACTH between ICI-aH and ICI-aA diagnosis with recovery of central adrenal insufficiency [18]. In our patient, we relied on other laboratory abnormalities: potassium was elevated and plasma renin activity was high, while aldosterone was low, suggesting primary aldosterone deficiency. Renin elevation was notable given his previous nephrectomy. The absence of 21-hydroxylase autoantibodies did not decrease the likelihood of diagnosing ICI-aA.

Adrenal imaging, such as CT and MRI, may support ICI-aA diagnosis, although data are scarce. Guidelines recommend performing a CT scan if adrenalitis is suspected to rule out metastases and hemorrhage [13]. Although no specific radiological pattern has been identified as typical of ICI-aA, global enlargement may be seen in the acute phase, progressing to glandular shrinkage in the chronic phase, as in our patient. This shrinkage is probably the result of prolonged corticosteroid use and relative ACTH suppression as well as parenchymal destruction [19].

In our case, since glucocorticoid replacement therapy for secondary adrenal insufficiency had already been started, we added mineralocorticoid supplementation, which improved the patient's symptoms, normalized potassium, and allowed hydrocortisone to be reduced to physiological doses. We cannot completely rule out that primary adrenal insufficiency was already present at the initial presentation, at the time of the hypophysitis diagnosis. However, due to severe headaches, the patient began glucocorticoid therapy before any laboratory investigations were performed. The subsequent development of new-onset hyperkalemia, changes observed in adrenal imaging, and the timeline of events (adrenalitis occurring at 30 weeks vs 9-12 weeks for hypophysitis) support the hypothesis that adrenalitis developed after the onset of hypophysitis.

Ultimately, we believe that liraglutide treatment delayed the diagnosis of adrenal insufficiency, as the patient's weight loss was attributed to the medication rather than to the underlying adrenal condition. There is no evidence to suggest that liraglutide can cause adrenalitis.

Recovery from ICI-related hypopituitarism differs from other causes of hypophysitis. Long-term hormone replacement is more likely to be required in patients with ICI-induced hypophysitis than in those with primary hypophysitis [8]. ACTH recovery is extremely rare [6, 13], while thyroid and gonadal function recovery occurs in up to 90% and 60% of the cases, respectively [6, 20]. Most recovery occurs within the first 3 months [6, 20]. In our patient, testosterone levels returned to normal at the last follow-up, indicating recovery of the gonadal axis. Regarding levothyroxine, it may be useful to continue to assess his thyroid function to determine the need for continued thyroid hormone replacement therapy. No ICI-aA recovery has been reported in the literature [15]. However, it remains beneficial to periodically reassess his fludrocortisone therapy by monitoring serum potassium levels and blood pressure.

## Learning Points

- Hypophysitis occurs in 10% of patients on anti-CTLA-4/PD-L1 therapy; adrenalitis is rarer (2%). Concomitant hypophysitis and adrenalitis are scarcely reported.
- At ICI-aH diagnosis, pituitary MRI may be normal or show gland enlargement, posterior enhancement, or stalk thickening. While no specific imaging pattern defines ICI-aA, bilateral adrenal thickening is typical.
- In cases of persistent symptoms despite corticosteroid dose adjustments, peripheral adrenal insufficiency should be considered, especially with hyperkalemia, high renin, and low aldosterone. ACTH is unreliable due to central suppression.

## Contributors

All authors made individual contributions to authorship. A.N., S.L., P.T., and M.M. were involved in clinical diagnosis and management. S.C. interpreted each radiological exam. A.N., S.L., and M.M. were involved in draft writing and manuscript submission. P.T. and S.C. contributed to manuscript revision. All authors reviewed and approved the final draft.

## Data Availability

Data sharing is not applicable to this article as no datasets were generated or analyzed during the current study.
